# Development and validation of the Body Compassion Questionnaire

**DOI:** 10.1080/21642850.2021.1993229

**Published:** 2021-11-21

**Authors:** Emily S. Beadle, Alison Cain, Shazia Akhtar, Joyce Lennox, Lauren McGuire, Nicholas A. Troop

**Affiliations:** Department of Psychology and Sport Sciences, University of Hertfordshire, Hatfield, UK

**Keywords:** Body compassion, eating behaviour, body image, self-compassion

## Abstract

**Background:**

The associations between compassion, self-compassion, and body image are well established. However, there is not yet a compassion-informed measure of *body compassion* that can be applied to any aspect of one’s body.

**Method:**

Items for The Body Compassion Questionnaire (BCQ) were derived from an earlier expressive writing study on self-compassion in body image. In study 1, the BCQ was completed by 728 men and women; with factor analysis, Rasch analysis, content and concurrent validation and reliability assessed. Study 2 compared BCQ scores with investigator-based ratings of spontaneous expressions of body compassion through writing in female undergraduates as well as an existing measure of body compassion. Study 3 examined the associations between BCQ scores, and the emotions expressed in a structured body image writing task. It also examined the relative predictive ability of the BCQ versus self-compassion in predicting eating pathology.

**Results:**

A bi-factor structure was identified, with an overall BCQ score and three subscales: body kindness, common humanity, and motivated action. The BCQ and its subscales had good validity and reliability and Rasch analysis showed the item fit was invariant across a range of demographic characteristics. Spontaneous expressions of body compassion showed positive associations with body kindness. Overall BCQ scores and body kindness were also inversely related to negative emotions expressed in relation to body image. The BCQ was a better predictor of eating disorder symptoms than was self-compassion.

**Conclusions:**

The BCQ is the first measure of body compassion that is aligned with theoretical aspects of self-compassion, and which includes aspects of both the first and second psychologies of compassion. It also highlights its potential use as a process measure of body compassion in models of eating disorder symptomology, mood and wellbeing as well as an outcome measure for compassion-based interventions in eating disorders and body image.

While many studies have demonstrated the importance of self-compassion in relation to physical and mental health outcomes, recent research on self-compassion in body image has identified *body compassion* as a potentially important construct. This report develops a measure of body compassion that improves on current measures and demonstrates its potential usefulness in relation to a range of health behaviours and mental health outcomes.

Compassion has been defined as ‘a sensitivity to suffering in self and others, with a commitment to try to alleviate and prevent it’ (Gilbert, 2014, p. 19). It has also been suggested that compassion is composed of four components (Jazaieri et al., 2013):

Cognitive – an awareness of suffering
Affective – sympathy with or being moved by suffering
Intention – desire to see relief of suffering
Motivation – responsiveness to relieve suffering

Both of these definitions incorporate two mind-sets that have been termed the psychologies of compassion (Gilbert, 2009, 2017a). These are the motivated sensitivity to suffering and motivated action to alleviate and prevent suffering. Gilbert (2009, 2017a) proposed six competencies to engage with suffering: sympathy, distress tolerance, empathy, non-judgement, care for wellbeing and sensitivity. Gilbert has also proposed six skills to alleviate and prevent suffering: helpful attention, imagery, reasoning, behaviour, sensory and feelings.

Gilbert (2017b) details the ‘flow of compassion’ (p. 44) from compassion we feel *for* others, openness and responsiveness, to compassion *from* others and finally to the capacity for *self-compassion*.

Building on this, Neff (2003a, 2003b) has defined self-compassion as being open to and touched by one’s own suffering and a desire to alleviate this to heal with kindness. Neff (2003a, 2003b) suggests there are three bipolar components to self-compassion: self-kindness as opposed to self-judgement, common humanity as opposed to isolation and mindful awareness (mindfulness) rather than over-identification of painful thoughts and feelings. The self-Compassion Scale (SCS: Neff, 2003b) has these 6 components (Self-kindness, common humanity, mindfulness, self-judgement, isolation and over-identification) as subscales which combine to form a single overall score or can be used as separate subscale scores to indicate these separate elements of self-compassion. More recently there has been dispute over the structure of the SCS with recent studies proposing a bi-factor model where all items load onto a single global measure of self-compassion directly as well as the six individual subscales (Neff, Whittaker, & Karl, 2017; Tóth-Király, Bőthe, & Orosz, 2017). This informs the analysis of the structure of the new measure of body compassion (see below).

## Theoretical rationale for a new measure of body compassion

The theory behind compassion is from evolutionary psychology and involves an affect regulation system and the three systems that are proposed to operate within it (Depue & Morrone-Strupinsky, 2005): the threat prevention system, the drive system and the contentment system.

The threat prevention system is designed to notice threats to the self and trigger emotions (e.g. anger). This elicits an appropriate behavioural response (e.g. fight, flight or submission) (Gilbert, 2001). However, because this threat prevention is over cautious, taking a better-safe-than-sorry approach (Gilbert, 1998a) it can be a source of psychopathology (Gilbert, 1998b, 2009), creating anxiety when recognising something as a threat when it is not. It has been theorised that early life events might sensitise this system to develop strategies to operate in certain situations to combat threats to the self. However, these can be maladaptive and lead to an increased vulnerability to anxiety or depression (Gilbert, 2009).

The drive system involves motivation for resources and or to reach goals. It is a source of anticipation and pleasure, however not necessarily happiness due to the dependence on reward and achievement (Gilbert, 2009). Status seeking, competitiveness and rejection avoidance have all been associated with this drive system (Depue & Morrone-Strupinsky, 2005).

The contentment system or social safeness system is associated with soothing, calm and positive affect and wellbeing, not simply the absence of threat. It is associated with attachment, the evolution of which led to signals of caring and kindness to be soothing and activate these positive effects (Depue & Morrone-Strupinsky, 2005; Gilbert, 2009). The contentment system is said to be a regulator of the other systems and as such is a key element in compassion-based therapies and the ability to self-soothe.

The balance of these systems is the foundation of compassion-based interventions for shame and self-criticism like compassion focused therapy (CFT e.g. Boersma, Håkanson, Salomonsson, & Johansson, 2014; Gilbert, 2009). The strategies for threat prevention and for attaining goals are associated not only with the basic emotions such as anxiety, anger, fear and disgust but also associated with self-conscious emotions, like shame (Tracy & Robins, 2004). Specifically, self-conscious emotions are associated with social situations and the achievement of social goals like status or to prevent rejection. It has been suggested that for women high in shame and criticism, disordered eating and weight management are a consequence of shame and self-criticism (Goss & Gilbert, 2002) and the association between body image, eating pathology and shame in community and patient groups has been demonstrated by a number of authors (e.g. Gee & Troop, 2003; Goss & Allan, 2010; Troop, Allan, Serpell, & Treasure, 2008). However, self-compassion has been suggested as an alternative to regulating threat and negative affect (Gilbert, 2009, 2017a, 2017b), such that it would replace these maladaptive strategies.

There is a wealth of literature supporting an association between body image and self-compassion. Although much of this is in young female North American samples (Kelly & Stephen, 2016; Raque-Bogdan, Piontkowski, Hui, Ziemer, & Garriott, 2016; Toole & Craighead, 2016; Wasylkiw, MacKinnon, & MacLellan, 2012), there is also evidence in females of all ages (Albertson, Neff, & Dill-Shackleford, 2015; Homan & Tylka, 2015), and in both male and female students (Rodgers et al., 2017, 2018). While self-criticism mediates the effect of early shame or abuse on disordered eating and body dissatisfaction (Dunkley, Masheb & Grilo, 2010; Gois, Ferreira & Mendes, 2018), the effect of current shame on binge eating disorder is also mediated by self-criticism (Duarte & Pinto-Gouveia, 2017).

The concept of *body compassion* or *body self-compassion* has been floated over the last decade, emerging as a theme in qualitative work in yoga intervention (Clancy, 2010), physical activity in paraplegic men (Smith, 2013), young women exercisers (Berry, Kowalski, Ferguson, & McHugh, 2010) and postpartum women (Woekel & Ebbeck, 2013). However, it has only recently begun to be explored and defined formally. Murn (2013) was the first to give a definition of body compassion as reflecting self-kindness, common humanity and mindfulness to one’s own body compared to judgemental, critical, isolating and over-identification with negative feeling and emotion. Body compassion was also described by Tylka and Wood-Barcalow (2015) whereby they suggest that self-compassion might promote body compassion through buffering the distress that can be caused by body-image related threats. Bringing together these definitions of *body compassion* with Neff’s (2003a, 2003b) *self-compassion* and Gilbert’s (2010, 2017a) *compassion*, *body compassion* can be described as compassion directed to one’s own body. It incorporates elements of kindness, common humanity and mindful awareness, as well as elements of sensitivity to body-related distress, pain and suffering as well as the motivation and ability to combat this.

Recently Altman, Linfield, Salmon, & Beacham (2020) described the development of a Body Compassion Scale (BCS) based around Cash’s (2002) definition of body image and Neff’s (2003a, 2003b) self-compassion. Cash’s definition of body image considers attitudinal dispositions towards the physical self, includes evaluative, cognitive and behavioural components, and includes appearance, competence, fitness and health or illness. However, this could be seen as contradicting Neff’s conception of self-compassion due to the *evaluative* component. By definition, *evaluation* involves some comparison to one’s own internal standards to one’s previous or desired achievements or to an ‘ideal’. Comparison to an unattainable, unrealistic or impossible ideal (Thompson, Heinberg, Altabe, & Tantleff-Dunn, 1999) in the Western world typically involves a slim/thin, youthful feminine ideal in women (Leonhard & Barry, 1998) and a lean, muscular masculine ideal in men (Pope et al., 2000). The feminine ideal has been shown to be implicated in eating disorders (Leonhard & Barry, 1998; Thompson & Stice, 2001; Thompson et al., 1999) with maladaptive perfectionism appearing to impact body image and eating behaviour through negative self-evaluations (Barnett & Sharp, 2016). *Compassion* rather than *changing self-evaluations* (as cognitive–behavioural therapies often do) focuses instead on *changing people’s relationships to self-evaluation* (Leary, Tate, Adams, Batts Allen, & Hancock, 2007).

The BCS also cements itself with mindfulness and acceptance-based (MAB) approaches which is clear from the *defusion* and *acceptance* subscales of the measure. This raises the question of whether it can really be considered ‘compassion’ as it appears to have more in common with mindfulness. Although these two concepts are certainly related (Germer & Barnhofer, 2017; Germer & Neff, 2013; Neff & Germer, 2013), and one can have mindfulness with compassion training and compassion within mindfulness training, a distinction can nevertheless be drawn. Mindfulness focuses on the experience while compassion focuses on the experiencer, with compassion being more emotionally activating than mindfulness and compassion training being uniquely able to help with shame (Germer & Barnhofer, 2017; Gilbert & Procter, 2006).

## Psychometric strategy

For the reasons above, the present paper describes the development and validation of a new measure of body compassion, informed by compassion, removing the focus on evaluation and on specific elements of one’s body and instead focusing on one’s feelings and thoughts of any part of one’s body. The development used a combined inductive and deductive approach or hypothetico-deductive approach (Walliman, 2018). The items were in part generated from expressive writing of people writing about their bodies, and as such are spontaneous expressions of self-compassion towards one’s own body. This was from an inductive approach; where the items measuring body compassion were from previous observation and analysis of the compassionate thoughts and feelings of these participants (Collis & Hussey, 2014; Janzen, Nguyen, Stobbe, & Araujo, 2015; Oosterveld, 1996). This scale development also considered a deductive approach or theory-driven approach (Collis & Hussey, 2014; Janzen et al., 2015; Oosterveld, 1996). This new scale, the Body Compassion Questionnaire (BCQ) incorporates elements of Gilbert’s (2009, 2010, 2014, 2017a) and Jazaieri et al.’s (2013) definitions of compassion and Neff’s (2003a, 2003b) self-compassion. Therefore, the theories of compassion and self-compassion were used to inform and refine the inductively formed items. Additionally the inductively formed items were themselves founded on the theory of self-compassion, as the participants were asked to write about their bodies considering first self-kindness, then common humanity and finally mindfulness (Neff, 2003a). Items were designed such that each item can be viewed in relation to any aspect of the body (not just weight and shape, health or function). The use of the BCQ is described in relation to disordered eating and mood in order to demonstrate the breadth of its potential uses.

Although no specific predictions are made, differences between men and women are also explored, both in terms of differences in means but also in terms of differences in correlations between scales and other relevant outcomes.

This scale development will also consider both classical test theory (CTT) and modern test theory (MTT; or item test theory) (Rusch, Lowry, Mair, & Treiblmaier, 2017; Magno, 2009). Therefore, in addition to the CTT incorporating factor analysis, reliability and validity testing that will be detailed below, this study considered MTT models that focus more at item level. MTT models are nonlinear and relate respondent performance on an item to the estimated level of the latent trait of interest (Urbina, 2005). These models are also assumed to be invariant across populations. Differential functioning and model fit can be assessed along with the functionality of the Likert scale and individual responses to items (Kline, 2005).

## Reliability and validity

This paper assesses the reliability and validity of the BCQ in a number of ways. Here a brief summary of the reliability and validity assessments is described, to be elaborated on in the methodology of each relevant study.

The reliability of the BCQ scores was assessed in study 1 through internal consistency (Kline, 1993) and external/test-retest reliability (Johnson, 2001; Rattray & Jones, 2007).

The validity was initially assessed using content validity (Rusticus, 2014), ensured through examination and ratings of the original 90 items of the BCQ and further examination of the 55 items of the BCQ by experts in self-compassion and compassion (with expertise in clinical psychology and compassion focused therapy as well as health psychology with research interests in self-compassion and compassion) (Hughes, 2018; Rattray & Jones, 2007). Criterion validity considers how well the scale correlates with or predicts another measure of interest (Piedmont, 2014b; Salkind, 2010). Here concurrent validity, a cross-sectional comparison (Lin & Yao, 2014), with eating disorder symptoms and body image avoidance behaviour has been considered. It was expected that, due to the previously shown associations between body image, self-compassion, eating disorders and body image avoidance (Braun, Park, & Gorin, 2016; Ferreira, Pinto-Gouveia, & Duarte, 2013; Kelly, Carter, & Borairi, 2014; Stapleton, McIntyre, & Bannatyne, 2016), that increased body compassion would be associated with reduced eating disorder symptoms and body image avoidance behaviour. In addition, it was expected, given the associations between body image, self-compassion and mood, for there to be associations between body compassion and mood. Predictive validity has also be considered in terms of incremental validity examining the effect of body compassion over and above self-compassion in predicting eating pathology.

Construct validity considers the extent to which a scale measures the theoretical construct it intends to (Ginty, 2013; Piedmont, 2014a). Cronbach and Meehl’s (1955) conceptualisation of construct validity outlined the need to clearly describe the relations between psychological processes or concepts and the theoretical reasons behind these (M. E. Strauss & Smith, 2009). As part of construct validity the importance of specifying the nomological network of the construct is frequently highlighted (Cronbach & Meehl, 1955; Leary, Kelly, Cottrell, & Schreindorfer, 2013). Table 1 demonstrates the investigated constructs and the hypothesised relationships. The theoretical and empirical reasons for these predicted directions are further described below. In addition, once the factor structure of the BCQ was established in study 1 a more detailed nomological network is described (see study 1 results).

**Table 1.** Hypothesised associations in studies 1–3 for construct validity, demonstrating the nomological network of body compassion.

Study	Correlates	Body Compassion
Study 1	Self-compassion (SCS)	+
	SCS-Self-kindness	+
	SCS-Common humanity	+
	SCS-Mindfulness	+
	SCS-Self-judgement	–
	SCS-Isolation	–
	SCS-Over-identification	–
	Body pride and shame (BPS)– current	–
	Body pride and shame (BPS)– gain	–
	Body pride and shame (BPS) – loss	–
	Depression-Happiness (SDHS)	+
Study 2	Body Compassion Scale (BCS)	+
Study 3	LIWC-Positive affect	+
	LIWC-Negative affect	–
	LIWC-Anxiety	–
	LIWC-Sadness	–
	LIWC-Anger	–

Note: – indicates negative relationship, + indicates positive relationship. SCS = Self-Compassion Scale, LIWC = Linguistic Inquiry Word Count, BCS = Body Compassion Scale, SDHS = Short Depression-Happiness Scale, BPS = Body Pride and Shame Scale.

Campbell and Fiske (1959) also considered particular elements of construct validity, namely convergent and divergent validity (M. E. Strauss & Smith, 2009). Convergent validity refers to the associations between constructs that are similar or the same as the tested measure (Chin & Yao, 2014; Ginty, 2013; M. E. Strauss & Smith, 2009). For example, body compassion would be expected to be associated with self-compassion, body shame and the BCS. Discriminant validity, by contrast, assesses the measure based on its association with concepts expected to be unrelated to the construct of interest (Ginty, 2013; Hubley, 2014). For example, a weak or non-significant association was expected between body compassion and age.

### Compassion and self-compassion

Items for the BCQ were generated from an expressive writing study where participants were asked to write about their bodies from a self-compassionate perspective, considering the 3/6 main components of self-compassion: self-kindness over judgement and criticism, common humanity over isolation and mindfulness versus over-identification (Neff, 2003a, 2003b). It was therefore assumed that elements of these components would form part of the factor structure of the BCQ and that the BCQ would be associated positively with self-compassion. Similarly, Gilbert’s (2009, 2010, 2017a) conceptualisation of compassion that considers compassion as applied to oneself or to others, entails two ‘psychologies’ of compassion. The first of these considers motivated sensitivity, engagement and appraisal of suffering to oneself or others. This considers elements of sensitivity, non-judgement, empathy, distress tolerance, sympathy and care for wellbeing. By contrast the second psychology considers motivated action to alleviate and prevent this suffering to oneself or others. It considers imagery, reasoning, attention, feeling, sense and behaviour. Similar to the elements of self-compassion forming the basis for the factor structure, it was expected that the associations between self-compassion and more general compassion that these elements of compassion would also help to inform the structure and theoretical basis for body compassion. It was also predicted (as indicated in Table 1) that overall self-compassion as well as the positive components of self-compassion would be positively associated with body compassion and that the negative components of self-compassion would be negatively associated with body compassion.

### Body pride/shame

Self-compassion has shown itself to be an important tool in combating shame including shame associated with one’s body (Ferreira et al., 2013; Mosewich, Kowalski, Sabiston, Sedgwick, & Tracy, 2011; Reilly, Rochlen, & Awad, 2014; Woods & Proeve, 2014). It was predicted (see Table 1) greater body compassion would be associated with less shame and more pride in one’s current body, while also being associated with less anticipated shame in losing or gaining weight.

### Affect and mood

Self-compassion has been shown to be associated with improvements in positive mood (Gilbert, 2009; Odou & Brinker, 2014) including in relation to body satisfaction and appreciation (Slater, Varsani, & Diedrichs, 2017). In addition shame and self-criticism have been shown to be associated with depression and negative affect (Gilbert & Irons, 2005). Associations have also been shown between body image and body shame and mood (Harper & Tiggemann, 2008; M Tiggemann & Kuring, 2004; Marika Tiggemann & Boundy, 2008). Given these associations it was expected that body compassion would be positively associated with mood, in that greater body compassion was associated with more happiness. In study 3 the associations with positive and negative affect words and with sadness, anger and anxiety related words in expressive writing would also be assessed. It was expected that body compassion would be positively associated with positive affect and negatively with negative affect, sadness, anger and anxiety.

## Study 1

The aim of this study was to test the preliminary validity of the 48-items of the BCQ. This study also aimed to explore the factor structure of the BCQ and to confirm whether a bifactor model is the best fit for the BCQ. Item fit, differential item functioning (DIF) and response categories were then also assessed. Additionally, it aimed to evaluate the internal consistency of the final factor solution and examine the BCQ’s association with psychological wellbeing measures.

### Method

#### Participants

There were 728 participants recruited online, through social media and online adverts, to take part in a questionnaire-based study on body image and physical activity. The participants received no reward, financial or otherwise for taking part in the study. There were 127 males and 592 females (9 stated other/rather not say) who took part. All participants were aged from 16 to 76 years (*M* = 28.38, SD = 11.92), with current BMI statistics ranging from 13.32-66.48 kg/m^2^ (*M* = 24.74, SD = 5.86). The majority of participants identified themselves as White British or European and the majority of participants were also from the UK or USA, most were single, had A levels or equivalent, and were in education (the majority full-time). There were 59 participants who indicated that they considered they had a disability. The summary of ethnicities, country of origin, marital status, education and occupation for each part of the study can be seen in Table 2.

**Table 2.** Demographic variables in Study 1 in EFA, CFA and test-retest samples.

		EFA (*N* = 364)	CFA (*N* = 364)	Test-Retest (*N* = 198)
Age Mean (SD)		28.78 (12.18)	27.83 (11.52)	32.30 (13.37)
BMI Mean (SD)		24.71 (5.69)	24.74 (5.97)	23.41 (4.22)
Males		66	61	14
Females		293	299	60
Other		3	2	0
Ethnicity				
White	British, Scottish, English, Welsh	191	175	47
	European	63	58	20
	American	17	12	0
	Australian	1	3	2
	Other	16	21	5
Asian	Chinese	9	12	3
	Indian	9	6	0
	Pakistani	2	1	0
	Filipino	2	1	0
	Singaporean	0	2	0
	Other	12	10	0
Black	African	6	13	0
	Caribbean	2	6	0
	Other	3	9	0
Other	Mixed Race	13	12	3
	Hispanic/Latino	6	10	0
	Native American	0	3	0
	Mexican	2	1	0
	Other	1	2	0
	Unstated	9	7	0
Dieting to lose weight		118	129	
Dieting to maintain weight		146	160	
Marital Status				
	Single	111	132	37
	Married/Civil Partnership	79	62	19
	Divorced	68	61	2
	Living with Partner	23	34	15
	In a Relationship	56	45	7
	Widowed	1	1	0
	Not Stated	26	29	0
Educational level				
	GCSE’s or equivalent	40	41	5
	A Level or equivalent	125	117	15
	Bachelor’s Degree	86	99	32
	Master’s Degree	45	39	23
	PhD or Higher	9	15	4
	None	33	23	1
	Not Stated	26	30	0
Job Role				
	Admin/Secretarial	31	41	8
	Professional	106	93	30
	Managerial	14	10	2
	Unemployed	17	10	2
	At home	10	10	3
	Self-Employed	10	15	4
	Studying/Education	150	156	31
	Not Stated	26	29	0

Note: EFA = Exploratory Factor Analysis sample, CFA = Confirmatory Factor Analysis sample.

Test-Retest: There were 198 participants from EFA/CFA (Confirmatory Factor Analysis) stages that gave contact details to be contacted for follow-up at four weeks. Of these, 83 participants completed the follow-up, however three of these had not completed sufficient baseline data to be of use here, leaving a final sample of 80 participants (40% uptake). Of these, 14 were male and 60 were female (6 other/unstated) and they were aged 16–69 (*M* = 32.30; SD = 13.37). Participants’ current BMI ranged from 14.77 to 37.22 (*M* = 23.41; SD = 4.22). The majority were White (74), with the rest Asian (3) or mixed race (3). Full breakdown of frequencies for ethnicity, marital status, education and job are shown in Table 2. The test-retest participants were significantly older than the original sample on average (*p* = .02), with significantly lower BMI (*p* = .029).

#### Measures – Body Compassion Questionnaire (BCQ)

Items for the *Body Compassion Questionnaire* (BCQ) were generated from an expressive writing study in which female students wrote about body image for 15 min per day for three consecutive days. One group about body image alone while another group wrote about body image from a self-compassionate perspective (day one focused on self-kindness over critical self-judgement, day two focused on common humanity rather than isolation and day three focused on mindfulness rather than over-identification). Items for the new measure were derived from the writing of the 44 participants in the body self-compassion group (mean age 20.8 (SD 5.7); mean BMI 21.kg/m^2^ (SD 4.1)). The instructions provided to participants were based on Pennebaker and Beall’s (1986) instructions on writing about trauma. Specifically, on Day 1 (self-kindness), participants were instructed:

- We would like you to write about the way you think and feel about your body. What you write is entirely up to you but write about the way you think and feel about your body in as much detail as you can. Really get into it and freely express any and all emotions or thoughts that you have about your body. *As you write, please think about the thoughts and feelings you describe and write in such a way that you express understanding, kindness and concern to yourself*. As you write, do not worry about punctuation or grammar, just really let go and write as much as you can in 15 minutes.

On Day 2 (common humanity), the italicised sentence above was replaced with ‘As you write, please think about the thoughts and feelings you describe and write in such a way that you consider how this is something that everyone may feel.’ On Day 3 (mindful awareness), the italicised sentence was replaced with ‘As you write, please think about the thoughts and feelings you describe and write in such a way that you are being realistic about your thoughts and feelings (i.e. neither denying nor exaggerating them).’

An initial pool of 90 items was then reviewed by four experts in compassion and self-compassion (a CFT practitioner and clinical psychologist, 3 health psychologists researching compassion and self-compassion) and reduced to 41 items. In this process items were removed on the basis they did not relate directly to a theoretically meaningful aspect of self-compassion, that they measured body image rather than body compassion and/or were ambiguous. Items were also re-worded, removing references to specific aspects such as weight or shape, so they could be applied to any aspect of one’s body (e.g. weight, height, function, health, appearance etc.). The final measure was formatted to ask participants to indicate how often they acted/felt in the manner stated in response to each item on a scale from 1 (almost never) to 5 (almost always). This format was chosen since it is also used in the Self-Compassion Scale (SCS: Neff, 2003b) and the Body Compassion Scale (BCS: Altman et al., 2020).

#### Measures – construct validation

The 26-item Self-Compassion Scale (SCS; Neff, 2003b) was used to measure self-compassion. This scale was developed to measure thoughts, emotions and behaviours associated with the subcomponents of self-compassion. It includes items on six subscales, three including positively worded items indicating the presence of compassion and three with negatively worded items indicating an absence of self-compassion (or the presence of self-criticism). The six subscales are *self-kindness* (SK) as opposed to *self-judgement* (SJ), *common humanity* (CH) rather than *isolation* (I), *mindfulness* (M) versus *over-identification* (OI). Responses are given on a 5-point scale indicating how often they behave in the stated manner where 1 = Almost Never and 5 = Almost Always. The SCS had an overall internal consistency of .91 (SK = .83, SJ = .85, CH = .76, I = .82, M = .78, OI = .77).

The Body Pride and Shame Scale (BPS; Troop, 2016) is a 30-item questionnaire used to measure behavioural, affective and attitudinal aspects of pride and shame. The degree to which these are experienced (or anticipated) in relation to current weight, imagined weight gain and imagined weight loss gives three subscales: BPS-Current, BPS-Gain and BPS-Loss. The 10 items for each of these three subscales are identical except for the temporal perspectives. Items are scored on 10-point Likert scales where 1 = ‘not at all true of me’ and 10 = ‘completely true of me’; high scores indicate more (current or anticipated) pride and low scores indicate more (current or anticipated) shame. Internal consistency of BPS-current was .91, for BPS-gain was .91 and for BPS-loss was .92.

The Short Depression-Happiness Scale (SDHS; Joseph, Linley, Harwood, Lewis, & McCollam, 2004) was used to measure depression and happiness. Developed from the 25-item Depression Happiness Scale (DHS; Joseph & Lewis, 1998), the SDHS includes three negatively and 3 positively worded items in order to maintain the bipolarity aspect of the DHS, where higher scores indicate greater happiness and lower depression, while lower scores indicate greater depression and lower happiness. Items are scored on a 4-point scale indicating that the person has ‘never’, ‘rarely’, ‘sometimes’ or ‘often’ felt in the stated way in the last 7 days. Internal consistency of the SDHS was .88.

#### Measures – concurrent validation

A brief version of the Eating Disorder Examination Questionnaire (EDE-Q; Fairburn & Beglin, 1994), assessed eating pathology. Grilo, Reas, Hopwood, and Crosby (2015) developed a seven-item version assessing three subscales: *Dietary Restraint* (*α* = .90)*, Shape and Weight Overvaluation* (*α* = .93)*, and Body Dissatisfaction* (*α* = .87). The three items of the dietary restraint subscale are assessed on a 0–6 Likert scale, where participants are asked for each item to rate ‘on how many of the past 28 days … ’, where 0 = 1–5 days, 1 = 6–12 days, 2 = 13–15 days, 3 = 13–15 days, 4 = 16–22 days, 5 = 23–27 days and 6 = every day. The shape and weight overvaluation and body dissatisfaction subscales are similarly assessed on a 0–6 point Likert scale but this time participants are asked to rate each item based on ‘over the past 28 days … ’, where 0 = not at all and 6 = extremely. Total EDEQ was computed by calculating an overall mean of the three subscales (as in the full version) and the overall internal consistency was .77.

The Body Image Avoidance Questionnaire (BIAQ; Rosen, Srebnik, Saltzberg, & Wendt, 1991) was used to measure the behavioural tendencies that accompany body image concern. This was created from interviews about what changes young women have made in their day-to-day routines as a result of body dissatisfaction and the changes this dissatisfaction had on their behaviour. Answers reported by at least three individuals were used to create a 19-item scale rated on a six-point (5-0) scale where 5 = always, 4 = usually, 3 = often, 2 = sometimes, 1 = rarely and 0 = never engaging in the listed behaviour. The BIAQ had an internal consistency of .84.

#### Procedure

Data were collected online through the survey engine Qualtrics in English. Participants were given basic information on the aims of the surveys and asked to give their consent to take part. They were then taken through the six questionnaires listed above as well as asked to provide basic demographic information about themselves. The procedure took approximately 30 min to complete and then participants were debriefed. Participants were also invited to complete a four week follow-up. Participants who agreed to be contacted in the follow-up and gave a contact email address were contacted four weeks after their initial participation with a link to the follow-up questionnaire (which included the BCQ amongst other measures) and a reminder of their anonymity number.

#### Ethics statement

This study was approved by the University of Hertfordshire, Health, Science, Engineering and Technology (previously Health and Human Sciences) Ethics Committee with Delegated Authority (ECDA).

#### Data analysis

SPSS 26 (SPSS Inc., Chicago, IL, USA) for the exploratory factor analysis (EFA) of the BCQ and SPSS Amos 23 (SPSS Inc., Chicago, IL, USA) was used to conduct the confirmatory factor analysis (CFA). For the CFA fit indices, Root Mean Square Error of Approximation (RMSEA) has been suggested to be the most informative criteria (Byrne, 2001), with values of < 0.05 (Browne & Cudeck, 1992) or <0.06 (Hu & Bentler, 1999) being suggested as indicative of a good fit, while 0.08 or less indicative of an adequate fit (Hair, Black, Babin, & Anderson, 2014). In addition to this the Comparative Fit Index (CFI) with values of >.90 and Incremental Fit Index (IFI) with values approaching 1.00 were also considered (Hair et al., 2014; Bentler, 1990; Bollen, 1989; Byrne, 2001) along with a TLI of >.90 (Hair et al., 2014).

Pearson’s *r* correlations were used to provide evidence of concurrent validity and intraclass correlations for test-retest reliability. Values of .10 would indicate low correlational effect, values of .3 medium and values of .5 a large effect (Cohen, 1988; Ellis, 2010).

Given the unequal sizes of males and females, effect size for the comparisons between these groups use Hedges’ g, where a small effect size is indicated by values >.20, a medium effect size indicated by values >.50 and large effect size by values >.80 (Ellis, 2010).

A Multi-dimensional Rasch analysis was conducted in WINSTEP version 4.6.20. Two main types of analysis were conducted to check if the items in the scale fit the model’s expectation; item fit and differential item functioning (DIF) e.g. (Lord, 1980; Wang, Yao, Tsai, Wang, & Hsieh, 2006; Wright & Stone, 1979).

In terms of item fit analysis – mean square statistics (MNSQ) were computed to determine item fit to the model. The MNSQ statistics show the amount of distortion of the scale. High MNSQ values indicate unpredictability and a lack of construct similarity with other scale items – this is referred to as underfitting (Wright, Linacre, Gustafson, & Martin-Lof, 1994). Low items show item redundancy and less variation in the data – this is referred to as overfitting (Wright et al., 1994). For the purposes of the present study, an accepted range of 0.7–1.2 (Wang et al., 2006) was used to identify items with poor model fit.

In terms of differential item functioning (DIF) – DIF analysis identifies items that appear to be too difficult or too easy, after having controlled for differences in the latent trait levels of the reference and focal groups. There were 4 main demographic characteristics of our participants; gender (2 groups: Males and Females), education (classified as 6 groups: GCSE, A’levels, Bachelor, Masters, PhD and None), age (classified as 5 groups: 16–29, 30–39, 40–49, 50–59, and over 60) and ethnicity (classified as 5 levels: 1 = white, 2 = Asian, 3 = black, 4 = mixed and 5 = other). It was important for the scale to be usable by as many individuals as possible, so testing for differences between these groups, ensured that any items which were responded to differently could be removed. We compared differences in the overall item difficulties across gender, age, education and ethnicity. If a difference was found between males and females, or between any two of the 6 educational levels, 5 age group levels and 5 ethnicity levels the item was considered as exhibiting DIF.

A difference larger than or equal to 0.5 logits is a sign of substantial DIF (Wang et al., 2006). Once a DIF item was identified, it was removed from further analysis. The multidimensional form of the partial credit model was again fitted to the new data set. The analyses stopped when all the infit and outfit MNSQ statistics were located within the (0.7, 1.2) critical range and no DIF items were identified.

### Results and discussion

These data were split in half randomly using a random number generator and allocating each participant a number. Half of these (*N* = 364) were used to conduct EFA on the BCQ, while the other half were used for the CFA. There were no significant differences on any demographic factors between participants in the EFA and CFA samples.

Recommendations for sample size vary for EFA and CFA. For factor analysis recommendations of a least 100 have been supported (Gorsuch, 1983), others have suggested at least 200 (Guilford, 1954) or 250 (Cattell, 1978) while Comrey and Lee (1992) stipulate 100 to be poor, 200 to be fair, 300 to be good, 500 to be very good and 1000 or greater to be excellent. Other recommendations have instead stipulated a ratio of 3:1 or 6:1 participant to variables (Cattell, 1978), while others suggest 20:1 (Hair, Anderson, Tatham, & Grablowsky, 1979).

For confirmatory factor analysis and structural equation modelling a minimum of 100 is required, but recommendations also vary in terms of the number of constructs being examined, communalities and under-identification of constructs (Hair et al., 2014). The sample size here of >300, would allow for 7 or fewer constructs, low communalities (.45) and/or multiple under-identified constructs.

#### Exploratory factor analysis (EFA)

Pearson’s *r* correlations were run to assess the items association with each other. It was found that some items correlated poorly (<.30) with all or most other items. As such items 13 and 22 were removed from subsequent analysis.

Factor analysis using the remaining 46 items was conducted with principal axis factoring. Kaiser-Meyer-Olkin of .919 and Bartlett’s sphericity were assessed: *χ*^2^(1035) = 8427.970, *p* < .001

**Figure 1.**
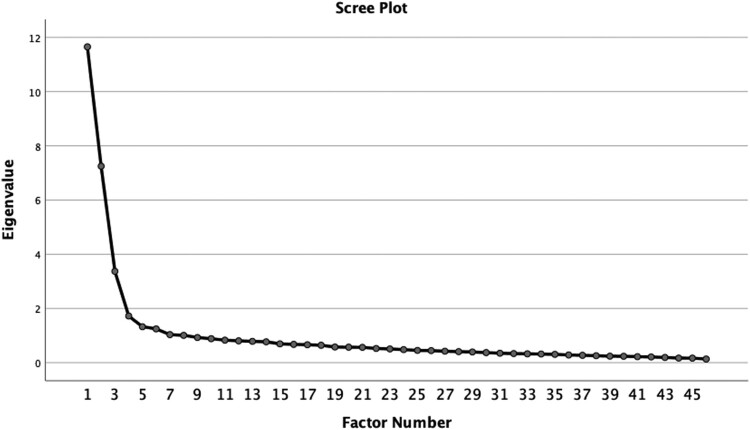
Scree plot showing the four-factor solution for the initial EFA.

From eigenvalues that were greater than 1, 8 factors were possible. However, on examination of the Scree plot (Figure 1) using the Cattell method, 4 factors were indicated.

Testing 4 factors with an Oblimin rotation produced factors that explained 47.63% of variance. Some items (7, 9, 15, 17, 18, 20, 26, 36, 37, 39, 40, 45, 46, 47, and 49) had poor loadings (<.5) and were removed (see Table 3). The factor analysis was then re-run without these items.

**Table 3.** Initial 4 Factor structure.

	Factor 1	Factor 2	Factor 3	Factor 4
50. I accept my body the way it is and an comfortable in my own skin	**.797**	–.001	.060	–.112
19. I do not really think a lot about my body, I accept this is me.	.**776**	.005	–.088	.042
11. I am happy in the body I have, no matter what size it is	.**768**	–.089	.065	.123
55. I feel quite comfortable in my body	.**768**	.038	.056	–.147
48. I feel ok with my body the way it is	.**762**	.033	.073	–.139
12. I have stopped worrying about weight and body shape	.**738**	–.069	–.095	.189
1. I like my body in spite of small inadequacies	.**712**	–.005	.127	–.170
41. I accept the flaws in my body, even if I don’t like them	.**710**	.008	.146	–.047
42. I am really grateful for the way my body is	.**692**	.009	.162	–.147
23. I am critical of my body’s flaws	–.**651**	.129	.184	.104
44. I am thankful for the way that I look	.**649**	.069	.223	–.149
21. I am critical of the way I think and feel about my body.	–.**577**	.061	.158	.275
54. It is hard to get away from the negative feelings I have about my body	–.**511**	.015	.104	.402
9. I do not want a different body image, I want to like the one I have.	.*469*	–.*029*	.*185*	–.*046*
39. Knowing that other people feel the same way about their bodies has really helped me a lot.	.*331*	.*123*	.*280*	.*172*
37. Knowing everyone feels the same does not make my insecurities about my body any less.	–.*326*	.*193*	.*065*	.*312*
28. I am sure everyone has insecurities about their bodies	–.072	.**787**	–.037	–.091
27. Everyone has something they do not like about their body	–.097	.**746**	–.101	–.169
33. Nearly everyone has some negative feelings about their bodies	–.105	.**744**	–.022	–.086
29. Everyone probably feels the same way about parts of their body that they would like to change.	.080	.**693**	.017	.064
25. Everyone has mixed feelings about their body	–.099	.**678**	.076	–.119
35. I think it is pretty normal to have hang-ups about certain parts of your body.	–.026	.**677**	.001	.083
31. I do not think anyone is completely satisfied with their body	.035	.**631**	–.172	.085
24. Body image is something that most people have issues with.	–.152	.**575**	.004	–.008
34. The way I feel about my body is probably a normal thing for everyone	.227	.**572**	.070	.118
30. There are people who have the same or even worse thoughts about their body image than I do.	–.004	.**524**	.111	–.212
36. The way I feel about my body is common amongst people I know.	.*159*	.*460*	.*065*	.*146*
26. My friends complain about the same things about their bodies as I do	–.*010*	.*331*	.*237*	.*117*
2. I am trying to become more accepting of my body	–.077	.067	.**657**	–.031
6. I try to empathise with myself and say I am ok and that I am happy with my body	.232	–.015	.**608**	–.064
4. I am working on making myself feel better about the way I look	–.212	.093	.**590**	.024
43. I need to be more accepting of my body	–.340	.053	.**582**	.155
3. I try my best to accept my body	.251	.011	.**574**	–.131
16. I try to be kind to myself about my body	.315	–.047	.**563**	–.151
45. I have positive as well as negative feelings about my body	.*183*	.*137*	.*471*	–.*293*
15. I tell myself that, even though I do not like my body, it is still capable of doing great things.	.*106*	.*032*	.*463*	.*106*
46. Although there are things I do not like about my body, I there are also things I do like	.*255*	.*192*	.*446*	–.*343*
40. Instead of thinking that I cannot like my body until it is perfect, I try and focus on the things that I do not like and try to love them.	.*336*	.*019*	.*445*	.*088*
7. I think I judge my body far too harshly	–.*380*	.*003*	.*428*	.*116*
49. I feel I can be too harsh on myself at times and need to accept my body	–.*204*	.*134*	.*409*	.*251*
47. Focusing on things I do not like about my body stops me thinking about all the good points and it makes me feel a lot more negative	–.*292*	.*017*	.*326*	.*261*
32. I often feel like the only person in the world with these thoughts about my body	.138	–.021	–.033	.**625**
38. I always feel alone in how negative my personal thoughts are about my body	–.267	–.006	–.075	.**617**
17. I really wish I did not hate my body because it is something that is always on my mind.	–.*463*	–.*008*	.*092*	.*498*
18. It is hard to accept that bodies are all different shapes and sizes	–.*207*	.*044*	.*046*	.*489*
20. I am starting to think that I worry about my body too much	–.*372*	–.*056*	.*267*	.*451*

This however left one factor with only 2 items as well as lower loading for item 15. The examination of these items revealed these were negatively worded common humanity items – e.g. feeling alone and isolated. Suggesting this was a separate component from the common humanity items suggested by factor 2.

**Figure 2.**
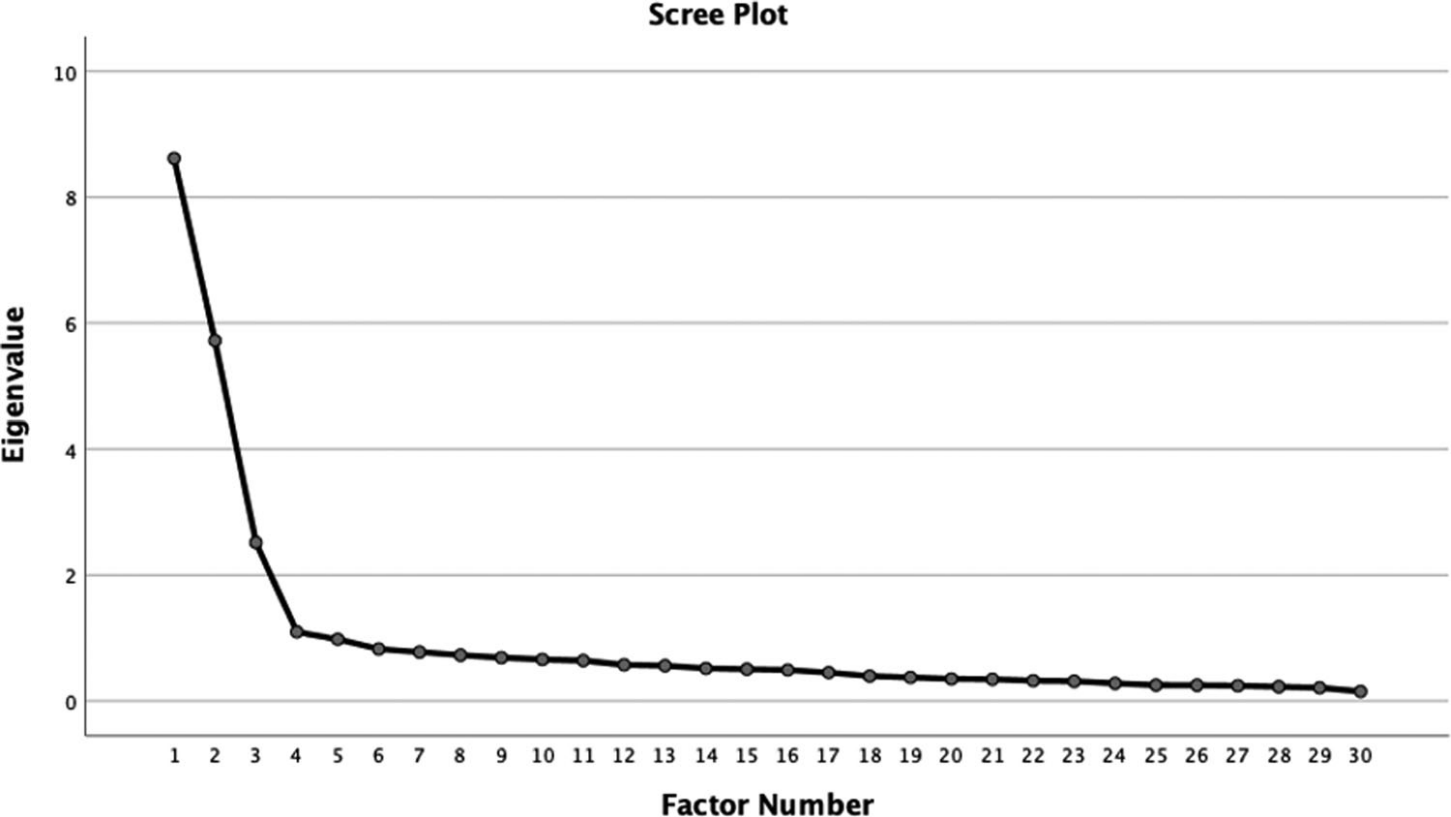
Scree plot showing the three-factor solution.

The factor analysis was therefore re-run without the factor 4 items and without items with low loadings (<.5). This indicated 3 factors, see Scree plot, Figure 2. As such this was rotated extracting 3 factors. Communalities can be seen in Table 4.

**Table 4.** Communalities for three-factor solution.

	Initial	Extraction
BC1	.696	.683
BC2	.531	.551
BC3	.561	.545
BC4	.442	.468
BC6	.434	.440
BC11	.581	.523
BC12	.504	.397
BC16	.496	.481
BC19	.565	.525
BC21	.597	.512
BC23	.605	.522
BC24	.415	.373
BC25	.525	.515
BC27	.597	.548
BC28	.647	.637
BC29	.486	.475
BC30	.342	.325
BC31	.358	.349
BC33	.552	.576
BC34	.418	.323
BC35	.506	.444
BC41	.592	.569
BC42	.695	.630
BC43	.417	.435
BC44	.665	.607
BC48	.706	.719
BC50	.776	.754
BC54	.524	.496
BC55	.785	.737

This 3-factor solution explained a total of 52.28% variance. The pattern matrix in Table 5 shows the item loadings onto each factor.

**Table 5.** Final 3-factor structure for the EFA.

	BK	CH	MA
50. I accept my body the way it is and an comfortable in my own skin	**.868**	.028	.021
55. I feel quite comfortable in my body	.**862**	.077	.008
48. I feel ok with my body the way it is	.**846**	.064	.039
1. I like my body in spite of small inadequacies	.**813**	.028	.091
42. I am really grateful for the way my body is	.**780**	.052	.094
44. I am thankful for the way that I look	.**743**	.115	.155
41. I accept the flaws in my body, even if I don’t like them	.**730**	.025	.129
19. I do not really think a lot about my body, I accept this is me.	.**724**	–.019	–.017
54. It is hard to get away from the negative feelings I have about my body	–.**708**	–.024	.159
21. I am critical of the way I think and feel about my body.	–.**704**	.038	.180
23. I am critical of my body’s flaws	–.**687**	.125	.169
11. I am happy in the body I have, no matter what size it is	.**685**	–.106	.127
12. I have stopped worrying about weight and body shape	.**610**	–.109	–.021
28. I am sure everyone has insecurities about their bodies	–.006	.**811**	–.049
27. Everyone has something they do not like about their body	.012	.**770**	–.129
33. Nearly everyone has some negative feelings about their bodies	–.045	.**757**	–.015
25. Everyone has mixed feelings about their body	–.037	.**679**	.097
29. Everyone probably feels the same way about parts of their body that they would like to change.	.056	.**676**	.049
35. I think it is pretty normal to have hang-ups about certain parts of your body.	–.073	.**648**	.024
31. I do not think anyone is completely satisfied with their body	–.012	.**615**	–.129
24. Body image is something that most people have issues with.	–.153	.**556**	.064
30. There are people who have the same or even worse thoughts about their body image than I do.	.120	.**541**	.075
34. The way I feel about my body is probably a normal thing for everyone	.141	.**529**	.089
2. I am trying to become more accepting of my body	–.065	.071	.**722**
4. I am working on making myself feel better about the way I look	–.225	.087	.**635**
3. I try my best to accept my body	.313	.013	.**633**
6. I try to empathise with myself and say I am ok and that I am happy with my body	.270	.002	.**577**
16. I try to be kind to myself about my body	.387	–.023	.**539**
43. I need to be more accepting of my body	–.396	.072	.**538**

Factor 1 (13 items) was named Body Kindness (BK), as it seems to reflect elements of kindness, acceptance and lack of judgement and criticism. Three items were negatively loaded, reflecting self-criticism. Factor 2 (10 items) was named Common Humanity (CH) and clearly reflects the idea of thoughts and feelings being shared by others. Factor 3 (6 items) was named Motivated Action (MA) and reflects the motivation to change the way one thinks, trying and working towards becoming more accepting, kind and empathetic with oneself.

#### Confirmatory factor analysis (CFA)

Bifactor models are models where correlations among items can be accounted for by a general factor representing a shared variance among the items and a set of grouping factors where variance is shared among items of similar content (Rodriguez, Reise, & Haviland, 2015). Each item should therefore load directly onto a general component as well as individual subscales. Although bifactor models have received less usage than higher-order factor solutions (Cucina & Byle, 2017; Reeve & Blacksmith, 2009), bifactor models have been suggested for the Self-Compassion Scale (Neff et al., 2017; Tóth-Király et al., 2017). The factors identified in EFA are seen as conceptually different subdomains but also with items expected to conform to the overall concept of body compassion vs. body criticism. This was similar to how the items of the self-compassion scale can be used in 6 individual factors or an overall concept of self-compassion. A bi-factor model would allow for the assessment of overall body compassion as well as subscale scores for each factor identified above.

In testing a bifactor model, the 3 subscales (BK, CH and MA) were loaded onto one side and overall score (body compassion) on the other, to assess the use of overall score as a component. The fit indices indicated fit was lower than ideal, with a CFI of .905, TLI of .888, GFI of .855 and RMSEA of .061 (*p* = .001). Examination of modification indices indicated the following items’ errors might be covaried to usefully improve the fit.

- Items 11 and 12, both of which relate to physique or body form
- Items 25 and 24 which both relate to ‘everyone’ being the same but in a way that feelings are mixed or neither positive or negative.
- Items 42 and 44 which relate to being thankful or grateful for their bodies
- Items 35 and 34 which relate to being ‘normal’
- Items 28 and 27 which relate to everyone feeling the equally as negative about their bodies
- Items 1 and 19 which relate to accepting of flaws
- Items 19 and 12 which relate to stopping worrying or thinking about their bodies

**Figure 3.**
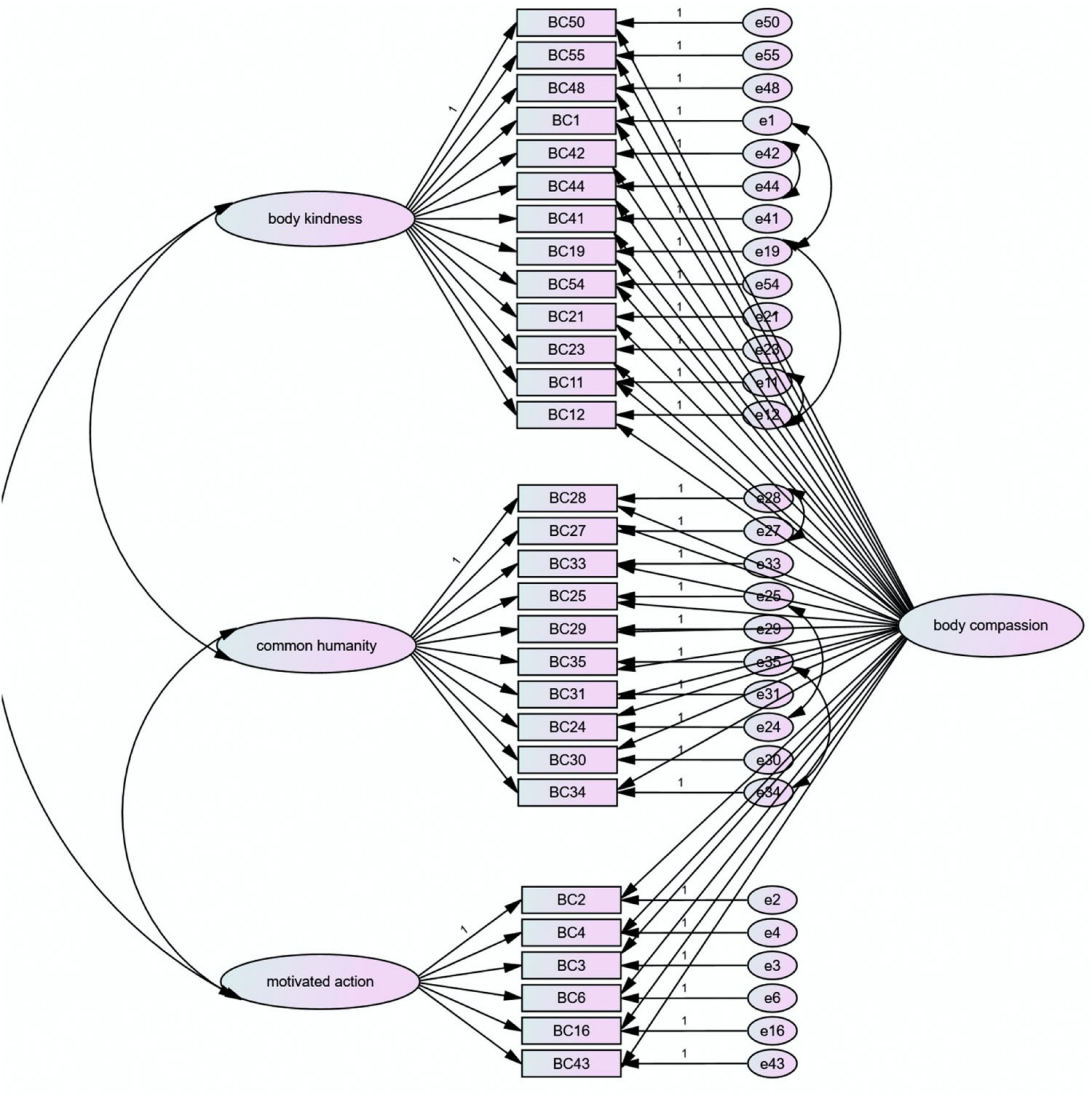
CFA Final factor model for the BCQ.

This model can be seen in Figure 3 and fit indices for the final model are in Table 6, showing superior fit, which reaches the threshold of good fit on key indices such as the CFI, TLI, IFI and RMSEA.

**Table 6.** Fit indices for final model.

Indices	Value
NPAR	97
CMIN	548.085
CMIN/DF	1.622
GFI	.895
AGFI	.895
NFI (delta1)	.883
RFI (rho1)	.860
IFI (delta2)	.952
TLI (rho2)	.941
CFI	.951
RMSEA	.044
RMSEA *p*	.914

### Response categories

The first analysis conducted was how respondents use the rating scale. In many cases respondents fail to react to a rating scale (Roberts, 1994). The Rasch analysis examines the average measure and threshold of each category. For the scale to be effective, we would expect observations in higher categories must be produced by higher measures. The average measures across categories must increase monotonically. In the present study, the 5-point scale and 29 items, the average measure increased with the category label (−0.14, 0.05, 0.21, 0.33 and 0.54) for categories 1–5 respectively. Moreover, threshold estimates also increased monotonically, logits of −0.66, - 0.3, 0.06, 0.35, 0.76. This suggests that the rating scale categorisation is satisfactory.

### Model data fit

Differential item functioning (DIF) analysis was conducted to assess the model data fit. None of the 6 items in the MA exhibited a substantial DIF. For the BK subscale, item 11 (I am happy in the body I have, no matter what size it is) exhibited substantial DIF between White and Asian, and between White and Black; item 21 (I am critical of the way I think and feel about my body) exhibited substantial DIF between males and females; Item 23 (I am critical of my body’s flaws) exhibited substantial DIF between males and females and between White, Asian and Black; item 54 (It is hard to get away from the negative feelings I have about my body) showed substantial DIF between males and females. Finally, for the CH subscale, item 29 (Everyone probably feels the same way about parts of their body that they would like to change) and item 35 (I think it is pretty normal to have hang-ups about certain parts of your body) exhibited substantial DIF for White, Asian and Black. These 6 items were deleted from the respective subscales, and the data were re-analysed. None of the remaining items exhibited substantial DIF. Table 7, shows maximum differences in the estimates for item difficulties. Moreover, the right had side of the table, shows the Infit and Outfit MNSQ statistics for the remaining 23 items. These values range from 0.8 to 1.29 where the acceptable range allocated was between 0.7 and 1.2. It is concluded that the 23 items fit the model’s expectation well.

**Table 7.** Maximum differences in the estimates for item difficulties (in absolute value) over Age, Gender, Education level, Ethnicity and infit and outfit MNSQ statistics.

Latent Variable	Questions	Age	Gender	Education	Ethnicity	Infit	Outfit
BK	1. I like my body in spite of small inadequacies	0.17	0.11	0.02	0.14	1.10	1.09
	11. I am happy in the body I have, no matter what size it is	0.09	0.16	0.19	0.64*		
	12. I have stopped worrying about weight and body shape	0.23	0.02	0.15	0.22	0.85	0.85
	19. I do not really think a lot about my body, I accept this is me.	0.34	0.15	0.25	0.41	1.16	1.19
	**21. I am critical of the way I think and feel about my body.	0.72*	0.39	0.07	0.32		
	**23. I am critical of my body’s flaws	1.12*	0.21	0.14	0.88*		
	41. I accept the flaws in my body, even if I don’t like them	0.43	0.33	0.17	0.11	1.05	1.01
	42. I am really grateful for the way my body is	0.22	0.03	0.23	0.14	1.00	1.02
	44. I am thankful for the way that I look	0.16	0.06	0.27	0.17	1.20	1.16
	48. I feel ok with my body the way it is	0.19	0.23	0.01	0.23	1.20	1.19
	50. I accept my body the way it is and am comfortable in my own skin	0.44	0.13	0.21	0.34	0.88	0.85
	**54. It is hard to get away from the negative feelings I have about my body	0.35	0.64*	0.14	0.29		
	55. I am quite comfortable in my body	0.22	0.19	0.16	0.11	1.08	1.04
CH	24. Body image is something that most people have issues with.	0.22	0.18	0.02	0.22	1.02	0.99
	25. Everyone has mixed feelings about their body	0.43	0.26	0.34	0.34	1.14	1.18
	27. Everyone has something they do not like about their body	0.44	0.17	0.25	0.45	0.86	0.86
	28. I am sure everyone has insecurities about their bodies	0.32	0.15	0.36	0.41	0.85	0.87
	29. Everyone probably feels the same way about parts of their body that they would like to change.	0.23	0.37	0.45	0.71*		
	30. There are people who have the same or even worse thoughts about their body image than I do.	0.42	0.42	0.39	0.33	1.19	1.18
	31. I do not think anyone is completely satisfied with their body	0.33	0.46	0.35	0.28	1.19	1.19
	33. Nearly everyone has some negative feelings about their bodies	0.46	0.17	0.33	0.33	1.19	1.17
	34. The way I feel about my body is probably a normal thing for everyone	0.48	0.01	0.29	0.39	0.84	0.84
	35. I think it is pretty normal to have hang-ups about certain parts of your body.	0.47	0.32	0.36	0.68*		
MA	2. I am trying to become more accepting of my body	0.12	0.09	0.05	0.32	0.80	0.82
	3. I try my best to accept my body	0.34	0.08	0.23	0.37	1.12	1.15
	4. I am working on making myself feel better about the way I look	0.29	0.07	0.25	0.15	0.83	0.87
	6. I try to empathise with myself and say I am ok and that I am happy with my body	0.42	0.08	0.15	0.22	0.99	1.01
	16. I try to be kind to myself about my body	0.69	0.07	0.36	0.29	1.18	1.15
	43. I need to be more accepting of my body	0.43	0.09	0.23	0.24	1.13	1.19

Note: *Substantial DIF (a difference in item difficulties larger than or equal to 0.5 logits between groups); Age 1 = 16–29, 2 = 30–39, 3 = 40–49, 4 = 50–59, and over 5 = 60;Gender = Males and Females; Education 1 = GCSE, 2 = A’Levels, 3 = Bachelors, 4 = Masters and 5= PhD, 6 = none; Ethnicity 1 = white, 2 = Asian, 3 = Black, 4 = Mixed and 5 = Other. **negatively worded item

Response category analysis was repeated on the 23 items. The average measures across the 5 response categories and threshold estimates increased monotonically, once again indicating the rating scale categorisation as satisfactory.

#### Internal consistency, descriptive statistics and inter-correlations

Mean scores were calculated for each subscale (*body kindness* [BK], *common humanity* [CH], *motivated action* [MA]) from the three-subscale, bifactor solution detailed in Figure 2. Additionally, an overall mean*body compassion* score (overall BCQ) was calculated.

Descriptive statistics for the BCQ are shown in Table 8. Internal consistencies indicated acceptable (≥.7) to excellent (≥.9) reliability in the scores.

**Table 8.** Descriptive statistics for BCQ scores.

	Min	Max	Mean	SD	α
BK (*N* = 641)	1.00	5.00	3.01	.97	.93
CH (*N* = 645)	1.13	5.00	4.00	.62	.84
MA (*N* = 643)	1.00	5.00	3.48	.77	.78
BCQ (*N* = 637)	1.52	4.96	3.48	.54	.86

Table 9 shows the means for the overall BCQ score and the subscales comparing males and females. Independent measures *t*-tests showed that females were significantly lower in BK (with a small effect size).than males. However, females were significantly higher than males in MA (with a medium effect size) and in CH (with a small effect size). There was no significant difference in overall BSQ scores.

**Table 9.** Gender differences for body compassion.

	Male	Female	*t*-Value	Hedges’ *g*
BK	3.30 (.94)	2.95 (.97)	3.31***	.36
CH	3.85 (.71)	4.03 (.60)	−2.31*	–.28
MA	3.15 (.83)	3.55 (.74)	−4.60***	–.53
Overall BCQ	3.46 (.51)	3.48 (.54)	.40	–.04

Note: ***p<.001. *p<.05

Intercorrelations between the subscales are shown in Table 10, which demonstrates good associations of all subscales with overall BCQ scores and significant (but with low *r*) associations between BK and MA and between CH and MA.

**Table 10.** Correlations between body compassion and variables relevant to construct validity.

	BK	CH	MA	Overall BCQ
CH	–.01	–	–	–
MA	−22*	.25***	–	–
Overall BCQ	.79***	.49***	.63***	–
BPS current	–.77***	–.06	–.15**	–.61***
BPS gain	–.64***	.10*	–.06	–.43***
BPS loss	–.17**	–.12*	–.24***	–.25***
SCS global	.55***	–.05	.10	.40***
SDHS	.48***	.13*	.16**	.45***
SCS-SJ	–.54***	.14**	.03	–.32***
SCS-I	–.35***	.18**	.08	–.15***
SCS-OI	–.37***	.20***	.10	–.15**
SCS-SK	.61***	.07	.33***	.56***
SCS-CH	.36***	.13*	.25***	.39***
SCS-M	.43***	.08	.21***	.41***
BMI	–.30***	.07	–.05	–.20***
Age	.09*	–.09*	–.24**	.06
EDE-Q	–.67***	.08	–.02	–.44***
BIAQ	–.58***	.03	–.06	–.45***

#### Construct validity

The predicted associations and directions for overall body compassion are shown in Table 1. Based on the factor naming processing and the theoretical and empirical reasons for these, predictions were made based on the factor structure shown earlier in this study.

*Body kindness* (BK) was predicted to be positively associated with SCS scores, in particular SCS-self-kindness (SK) with weaker associations predicted for SCS-common humanity (CH) and SCS-mindfulness (M). It was also predicted to be negatively associated with body pride and shame (BPS) (most strongly with the current – BPS scores) and with SCS-self-judgement (SJ), SCS-isolation (I) and SCS-over-identification (OI). It was also predicted that *body kindness* in particular would be positively associated with mood (SDHS).

*Common humanity* was predicted to be most strongly positively associated with the SCS subscale SCS-CH and less so with SCS overall, SCS-SK and SCS-M. It was predicted to be negatively associated with SCS-I and less so with SCS-SJ, SCS-OI, and BPS scales. It was also predicted that it would be positively associated with the SDHS.

*Motivated action* (MA) was predicted to be associated positively with overall SCS scores, SCS-SK, SCS-M and, SCS-CH to a lesser extent. It was also predicted to be negatively associated with SCS-SJ, SCS-I and SCS-OI as well as negatively associated with BPS subscales. It was also predicted that it would be positively associated with the SDHS.

The correlations between the relevant variables are shown in Table 10.

This shows that the predictions were correct for *overall BCQ scores* and for *body kindness.* For *common humanity*, *r* values were low for all variables, with moderate correlations between *motivated action* and SCS-SK, BPS-loss, SCS-common humanity and SCS-mindfulness.

In order to further investigate these associations, given the differences between males and females, the correlations were considered split by gender (Table 11). This shows that *body kindness* and *motivated action* were significantly associated in females only. SCS-CH and all BCQ variables were significantly correlated for females only (except for overall BCQ itself which was significant for both). The association between *motivated action* and SC-M was also only significant for females. By contrast SCS-SJ, SCS-I and SCS-OI were only associated with *common humanity* and *motivated action* in males. Finally, slight differences were present for SDHS where it was significant and moderately correlated with all BCQ variables in females but only overall BCQ and *body kindness* in males. Slight difference was also present for the association between BPS loss and BCQ scores: there were significant and low to moderate correlations with all BCQ variables in females, but only with overall BCQ and *body kindness* in males. BMI was also more strongly associated with *body kindness* and overall BCQ in females than males.

**Table 11.** Correlations split by gender.

	Male				Female			
	BK	CH	MA	BCQ	BK	CH	MA	BCQ
CH	–.18	–	–	–	.05	–	–	–
MA	.18	.21*	–	–	.27***	.24***	–	–
BCQ	.71***	.44***	.65***	–	.82***	.50***	.64***	–
BPS current	–.68***	–.01	–.14	–.55***	–.79***	–.08	–.20**	–.64***
BPS gain	–.66***	.12	–.20	–.52***	–.63***	.08	–.06	–.42***
BPS loss	–.29*	–.02	–.15	–.27*	–.16*	–.15*	–.24***	–.24***
SCS mean	.50***	–.19	–.01	.24*	.56***	.01	.18**	.45***
SDHS	.56***	.02	–.01	.41***	.47***	.17**	.22***	.46***
SCS-SJ	–.53***	.38**	.29*	–.11	–.54***	.05	–.08	–.38***
SCS-I	–.39**	.28*	.29*	–.04	–.34***	.14*	–.003	–.18**
SCS-OI	–.41***	.29*	.25*	–.07	–.35***	.15*	.02	–.17**
SCS-SK	.61***	–.04	.31**	.55***	.62***	.10	.33***	.57***
SCS-CH	.16	.07	.16	.21*	.42***	.15*	.29***	.44***
SCS-M	.41***	–.04	.01	.28**	.44***	.14*	.31***	.46***
BMI	–.21	.18	.06	–.05	–.32***	.05	–.08	–.24***
EDE-Q	–.66***	.13	.05	–.39**	–.66***	.05	–.08	–.46***
BIAQ	–.34**	.02	.07	–.21	–.61***	–.07	–.13*	–.50***

This suggests that the associations between the BCQ (*overall* and *body kindness* scores) and these other constructs are broadly consistent in both genders. However, *common humanity* and *motivated action* can act quite differently in males and females in their associations with these other constructs.

#### Concurrent validity

Concurrent validity was assessed through association between BCQ and EDEQ and BIAQ. In addition to the predictions made in Table 1 it was predicted that both BIAQ and EDEQ should be negatively associated with *body kindness, common humanity* and *motivated action* subscales. Table 10 shows the correlations between these, while Table 11 shows this split by gender. This shows that associations were as predicted for overall BCQ scores and for *body kindness*, but no significant associations were shown for *motivated action* or *common humanity*. When examined by gender, there are no major differences to be observed.

#### Test-retest reliability

Correlations between baseline and one month follow-up, showed good correlations for *body kindness* (*r*(38) = .93, *p* < .001), *common humanity* (*r*(38) = .67, *p* < .001), *motivated action* (*r*(38) = .64, *p* < .001) and overall BCQ (*r*(38) = .89, *p* < .001).

### Conclusions of Study 1

The results of Study 1 demonstrate that the BCQ was a bi-factor model whereby researchers can use the overall mean BCQ score and/or its three subscales; *body kindness*, *common humanity*, and *motivated action (see supplementary materials table 1 for full scale)*. Item fit was invariant across a range of demographic characteristics and the response option Likert scale was appropriate. Finally, the scale scores demonstrated good internal consistency, validity and test-retest reliability.

## Study 2

Study 2 further examined the validity of the BCQ by cross-validating it with spontaneous expressions of body compassion in text generated by participants when writing about body image. Since beginning collecting data for Study 1, another measure of body compassion has also been published, the Body Compassion Scale (Altman et al., 2020). Study 2 therefore also cross-validates the BCQ with the BCS.

### Method

#### Participants

As part of a larger study, 27 female psychology students participated in an expressive writing study for course credit. Participants had a mean age of 21.88 years (SD 7.05, ranged from 18 to 50). Participants were predominantly white (70.4%), A-level holders (85.2%) and single (63.0%).

#### Measures

In addition to the 23-item BCQ, participants also completed the Body Compassion Scale (BCS; Altman et al., 2020). The BCS aims to measure an individual’s compassion toward their body with factors including defusion, common humanity and acceptance. A high score on the BCS equates to a greater level of body compassion. The BCS has 23-items and is measured using a five-point Likert scale (1 = almost never believe it and behave in this way to 5 = almost always believe it or behave in this way). An example item is, ‘When I feel out of shape, I try to remind myself that most people feel this way at some point’. In the current study, the BCS total score showed a Cronbach’s alpha of .71, while defusion had an alpha of .95, common humanity had an alpha of .86 and acceptance had an alpha of .87.

#### Procedure

Participants were provided with a document explaining what the study entailed and were asked to sign a consent form. Questionnaires were completed electronically, except for the expressive writing task, which in all cases was completed on paper. After the questionnaires were completed, participants were presented with an envelope containing the writing task and worksheet. Participants were asked to complete a writing exercise about their body image. Specifically, participants were given the following instructions, based on those originally developed by Pennebaker and Beall (1986) and modified as shown:

We would like you to write about the way you think and feel about your body. What you write is entirely up to you but write about the way you think and feel about your body in as much detail as you can. Really get into it and freely express any and all emotions or thoughts that you have about your body. As you write, do not worry about punctuation or grammar, just really get into it and write as much as you can in 15 minutes.

Participants were timed to write for 15 min before being debriefed and provided with an information sheet with various helplines for mental health support.

#### Ethics statement

This study was approved by the Health, Science, Engineering and Technology (previously Health and Human Sciences) Ethics Committee with Delegated Authority (ECDA), University of Hertfordshire. As with study 1, all questionnaires and writing instructions were administered in English.

#### Data analysis

The texts were rated by EB and NT in terms of expressions of body kindness, common humanity, and motivated action. Ratings were made on a four-point scale where presence of body compassion statements were given as 1-*none*, 2-*some*, 3-*moderate* and 4-*marked*. The first five cases were used to develop the coding and the remainder to establish validity of the BCQ. The ratings for each coder were entered into SPSS 26 (SPSS Inc., Chicago, IL, USA) and then an agreement was calculated using intraclass correlation (agreement). Spearman’s Rho was used to assess the relationship between the coder ratings and the other measures described above including the BCQ. The BCQ correlations used Pearson’s *r* as in study 1. Missing data were excluded pairwise.

### Results and discussion

Means (SDs) for the BCQ for this sample were as follows: BCQ-overall = 3.69 (.55); BCQ-Body Kindness = 3.08 (.86); BCQ-Common Humanity = 4.27 (.71); BCQ-Motivated Action = 3.83 (.63). Means for the BCS were: BCS-Total = 75.26 (17.27); BCS-Defusion = 2.60 (1.23); BCS-Common Humanity = 3.23 (.82); BCS-Acceptance = 3.12 (.92).

Intra-class correlation for the agreement between raters on spontaneous expressions of body compassion was .76 for Body Kindness, .85 for Common Humanity, and .61 for Motivated Action. This shows moderate to good agreement on the proposed components of body compassion, albeit the agreement on Motivated Action is slightly lower than for other components. Where there were differences in the investigator ratings for the spontaneous expressions of body compassion, these were resolved by discussion and the agreed score was used in the remainder of the analyses. Correlations between investigator ratings for body compassion and participant scores on the BCQ are as follows: body kindness (*r*(27) = .51, *p* = .003); motivated action (*r*(27) = .26, *p* = .10); common humanity (*r*(27) = .04, *p* = .42).

Table 12 shows the correlations between the BCQ and the BCS. This shows that body kindness and all BCS subscales (except Common Humanity) and BCS-Total were significantly associated (defusion negatively so). It also shows that common humanity of both subscales were significantly associated, while overall BCQ scores were significantly correlated with all BCS subscales. BCQ-Motivated Action was not significantly associated with any of the BCS subscales except for BCS-Common Humanity. Nor was it significantly associated with BCS Total. This suggests the BCQ taps into a component that is not captured by the BCS.

**Table 12.** Correlations between BCQ and BCS scores.

	BCQ-BK	BCQ-CH	BCQ-MA	BCQ-global
BCS-Acceptance	.85***	–.05	.22	.45*
BCS-CH	.27	.54**	.55*	.45**
BCS-Defusion	–.63***	–.01	–.13	–.43*
BCS-global	.75***	.25	.21	.63***

### Conclusions of Study 2

Study 2 demonstrates preliminary findings that spontaneous expressions of body compassion, identified in text, are consistent with scores for BCQ body kindness. However, they are less consistent for common humanity and motivated action. It may be that motivated action and common humanity are harder to express spontaneously in writing or else harder to identify in written texts than they are in self-report. However, it must be considered that this may be due to the lower sample size, and as such future research should examine body compassionate writing ratings and scores of the BCQ in more detail in larger samples.

Scores on the BCQ were also broadly correlated with the BCS in terms of overall score and subscale scores. However, the motivated action subscale of the BCQ was not associated with the BCS or the acceptance and defusion subscales. Since motivated action reflects the second psychology of self-compassion, this may suggest that the BCQ has identified an important aspect of self-compassion that has been missed by the BCS.

## Study 3

Self-compassion has been suggested to be associated with eating disorders as well as body image. In particular it has been suggested to protect against eating disorders (ED) in 4 ways: directly affecting ED outcomes, affecting the initial occurrence of ED risk factors, interrupting the effects of ED risk factors, and/or disrupting the mediation chain that the ED risk factors operate with (Braun et al., 2016). The negative (critical) subscales of the SCS have been especially associated with disordered eating (James et al., 2016; Kelly & Tasca, 2016), with evidence also suggesting that while self-compassion predicts body dissatisfaction (Barnett & Sharp, 2016; Maraldo, Zhou, Dowling, & Vander Wal, 2016) body dissatisfaction predicts disordered eating (Maraldo et al., 2016).

For these reasons it was expected that body compassion would predict disordered eating better than self-compassion. It was also predicted that, when asked to write about body image, individuals with higher body compassion would express more positive emotions and less negative emotions than those with low body compassion (more body criticism).

The aims of Study 3 were (1) to examine the linguistic content of body image writing and the association with body compassion and (2) to examine the predictive strength of body compassion (using the BCQ) in comparison to self-compassion in predicting disordered eating.

### Method

#### Participants

Participants consisted of 45 female psychology students participating for course credit. Their ages ranged from 18 to 62 (*M* = 27.87, SD = 13.44). Participants were predominantly white British (66.7%), and single (35.6%) or had a partner (33.3%).

#### Measures

In addition to the 23-item BCQ, the following measures were used:

The Self-Compassion Scale, Short Form (SCS-SF, Raes, Pommier, Neff, & Van Gucht, 2011) was used to assess self-compassion. This scale includes 12 items designed to test self-kindness vs. self-judgement, common humanity vs. isolation, and mindfulness vs. over-identification. Participants were asked to rate the items on a 1-to-5 Likert scale as in the full version described in study 1. The short scale was used because it has been found to be as reliable as the full scale when looking at total scores and to see the association between body compassion and this short version of the SCS. The questionnaire had an internal consistency of *α* = .85.

The Eating-Disorder Examination Questionnaire, version 6.0, (EDE-Q, Fairburn & Beglin, 1994) was used to examine participants’ weight (WC), eating (EC) and shape concerns (SC) and dietary restraint (DR). Participants were asked to answer the questions in relation to the last 28 days. The higher the participants’ scores, the more indicative this is of disordered eating, as it highlights frequency to partake in behaviours associated with eating disorders. The internal consistency was *α* = .92 (DR = .84, EC = .78, SC = .88, WC = .83).

The Short Depression-Happiness Scale (SDHS; Joseph et al., 2004) was used to measure depression and happiness and was described in Study 1. Here the internal consistency was .73.

To stimulate writing about body image, participants completed a structured open-ended questionnaire developed by the YWCA Social Action and Advocacy Committee of the Waterloo Region. Questions asked participants to write about what self-esteem is, what body image is, how they might be related, to consider what factors influence body image and what they might change about themselves. Responses were typed up and analysed using the Linguistic Inquiry and Word Count (LIWC: Pennebaker, Booth, & Francis, 2007). The LIWC counts words and assigns them to various psychological processes including emotional, cognitive and social words and represents the use of these words as a percentage of the whole text. In the present study, only words relating to positive and negative emotions were examined (the category of negative emotion also includes subtypes of anger, anxiety and sadness).

#### Procedure

Participants were informed briefly about the outline of the study before signing up. They were then reminded of the nature of the study in more detail by an information sheet, and then were asked to complete a consent form once it was confirmed they fully understood the study. Participants then completed all questionnaires consecutively. The researchers were in the presence of participants at all times. Once all the forms were completed, participants were thanked, given a debrief sheet and a list of support resources should they need them. All materials were presented in English.

#### Ethics statement

Following ethical approval for the study (approved by the Health, Science, Engineering and Technology ECDA), participants were recruited from the University of Hertfordshire, participants signed up to complete the study for course credit.

#### Data analysis

As described above participants’ written texts were analysed using the LIWC, this was then converted into SPSS 26 (SPSS Inc., Chicago, IL, USA) along with the rest of the data from the questionnaires. Missing data were excluded pairwise and analysis by analysis.

### Results and discussion

The descriptive statistics and correlations for each variable are shown in Table 13. These are broadly consistent with Study 1 for the BCQ variables (for females as shown in Tables 8 and 10) and slightly higher than community norms for the EDE-Q (Fairburn, Cooper, & O’Connor, 2008; Mond, Hay, Rodgers, & Owen, 2006). However, EDE-Q scores are, in general, higher for a younger sample (Mond et al., 2006), as in this study.

**Table 13.** Descriptive statistics and correlations between BCQ, EDE-Q, SDHS, SCS-SF and word usage in study 3.

	Mean (SD)	BK	CH	MA	BCQ-overall
BK	2.90 (.81)				
CH	4.15 (.53)				
MA	3.37 (.58)				
BCQ-overall	3.46 (.37)				
EDE-Q	1.90 (1.33)	–.77***	–.08	.06	–.67***
SDHS	19.36 (2.96)	.14	.14	.24	.29
SCS-SF	2.86 (.69)	.41**	–.02	.05	.30*
Positive words	6.81	.04	–.02	–.03	.02
Negative words	1.44	–.42**	–.20	–.12	–.50***
Anxiety words	.43	–.01	–.22	–.10	.16
Anger words	.25	–.19	–.25	.04	–.27
Sadness words	.47	–.42**	.19	–.15	–.32*

BCQ overall score and *body kindness* (BK) were significantly negatively correlated with the use of negative emotion words, and sadness words in particular, in writing about body image.

The BCQ overall score was significantly and negatively correlated with EDE-Q scores, while the subscale of BK was also negatively correlated, as in study 1. BCQ scores were also correlated positively with SCS-SF scores but, of the subscales, only the BK subscale was significantly (positively) correlated. In terms of SDHS scores, there were no significant correlations in this sample.

Multiple regression was used to assess the ability of the BCQ to predict EDE-Q scores after controlling for the influence of self-compassion (SCS). SCS-SF scores accounted for 7.5% of variance in global EDE-Q, which was shown not to be significant (*p* = .07), while the addition of the BCQ added 37.7% of variance explained to a total of 45.30% (*F* change (1, 42) = 28.97, *p* < .001). In the final model (*F* (2, 42) = 17.38, *p* < .001), only the BCQ was a significant independent predictor (*beta* = -.64, *p* < .001).

The subscales of the BCQ and SCS-SF were also examined in predicting EDE-Q. The SCS-SF subscales (self-kindness, self-judgement, common humanity, isolation, mindfulness and over-identification) combined accounted for 31.5% of variance in EDE-Q, which was shown to be significant (*p* = .019). The addition of the BCQ subscales (BK, CH, MA), however added 31.0% variance for a total of 62.5% of variance accounted for; *F* change (3, 35) = 9.63, *p* < .001. In the final model (*F* (9, 35) = 6.48, *p* < .001), only BK was a significant independent predictor (*beta* = -.77, *p* < .001).

### Conclusions of Study 3

Study 3 shows that, in writing about body image, people with higher levels of body compassion use fewer negative emotion words overall and, in particular, fewer sadness and anger words. In terms of its subscales, people with higher levels of body kindness use fewer negative emotion words overall, and fewer sadness words in particular. Conclusions drawn are limited by the sample size. This should be explored in larger samples to test whether more effects might be found for body compassion and eating behaviour.

## General discussion

The present paper describes the development and validation of a measure of body compassion, the Body Compassion Questionnaire (BCQ).

### Findings

Study 1 indicated that the BCQ was a bifactor model whereby researchers can use the overall BCQ score and/or its three subscales: body kindness, common humanity, and motivated action. The measure showed good internal consistency and test-retest reliability and the item fit is invariant across a range of demographic characteristics. Across the studies reported, the BCQ has demonstrable concurrent validity in terms of its associations with measures of eating pathology and body avoidance behaviour as well as construct validity in terms of its association with measures such as self-compassion, body pride and shame, mood and emotions. It also has good content validity since items were generated by participants writing about body image with self-compassion, and then reviewed and screened by four researchers/clinicians experienced in self-compassion research. Study 2 also showed the validity of the BCQ in terms of associations with expert ratings of spontaneous expressions of body kindness. Associations with an existing measure of body compassion, the BCS (Altman et al., 2020), were generally as expected although the inclusion of a motivated action subscale in the BCQ appears to be unique. Study 3 showed that body compassion was negatively associated with the use of negative emotion words, especially sadness, in writing about body image. It also showed that body compassion in relation to body image was uniquely predictive of eating pathology while general self-compassion was not.

This study details elements of the nomological network of the BCQ including the associations with the constructs of body pride and shame and self-compassion as well as with mood. These were largely shown to be as expected. This initial exploration of the construct validity of the BCQ suggests it was strongly associated with self-compassion. This is backed-up by the strong associations with the theory of self-compassion (Neff, 2003a, 2003b), with items generated from texts where participants were asked to write about body image from a self-compassionate perspective from the three components of self-compassion: self-kindness, common humanity and mindfulness. The components of the BCQ also show strong theoretical associations with elements of self-compassion related to acceptance, emotional responses, sensitivity to suffering, mindful awareness, common humanity, criticism and judgement (Germer & Neff, 2019; Gilbert, 2017b; Neff, 2003a, 2003b; Neff & Knox, 2017)) as well as with the first and second psychologies of compassion (Gilbert, 2017b) and with the components detailed in the BCS (Altman et al., 2020). These studies also show that the BCQ was strongly associated with body pride and shame and may indicate the potential for body compassion to activate in response to body shame to help reduce feelings of criticism, isolation and judgement in favour of compassion. This may lead to more healthy wellbeing in terms of mood (also shown to be strongly associated with body compassion in study 1) and with eating and body image avoidance behaviours.

### Strengths and limitations

The studies reported here identified a theoretically and psychometrically sound measure of body compassion that was superior to existing measures and applicable to a range of outcomes and contexts. Items were generated by participants writing compassionately about their body image which also makes it likely that items in the BCQ are worded to reflect the actual experience of body self-compassion. In addition, the inclusion of Rasch analysis further strengthens the scale in terms of giving a greater awareness of the model fit, response categories and differential item functioning.

One limitation is that these items were generated from a study involving only female participants and not males. Additional or different items may have been generated by males. Nevertheless, items were deliberately written to minimise references to specific body-related content (e.g. shape, function) in order to minimise the degree to which this may be a gendered issue. It is of note that overall BCQ scores did not differ between men and women. However, further evaluation with males is warranted including examining measure invariance with sufficiently large samples.

Validity was demonstrated through a range of measures and methods including both self-report as well as behavioural, for example relating body compassion scales to objective ratings of spontaneous expressions of body compassion and to the use of emotion words identified by computerised text analysis in expressive writing tasks. Nevertheless, the research is not without limitations.

Both EFA and CFA demonstrated the factor structure of the BCQ, added to by the model fit demonstrated by the Rasch analysis, although further evaluation in more diverse samples is warranted. The lack of strong correlations between certain aspects of the BCQ should also be considered, though this did vary between studies. Scores on the common humanity subscale were not strongly related to ratings of spontaneous expressions of common humanity in written texts. Although the items for all subscales originated from people writing compassionately about body image, the relatively high scores of common humanity in the questionnaire may indicate the relative superficiality of this subscale. Specifically, participants readily endorsed items on the common humanity subscale of the BCQ but did not generally express such attitudes spontaneously in their writing. Another issue concerns whether body compassion is a state or a trait and, therefore, whether it is amenable to change and whether this change can be measured. Future work could examine this issue by modifying the instructions to participants to specify different time frames (e.g. *in the past week* or *right now*). While it may be possible for future research to improve the items in this subscale, it may simply be that a self-report measure is not a good way to differentiate some attitudes if they have become glib truisms (e.g. people generally acknowledging that everyone feels the same way without fully internalising this observation as an aspect of self-compassion). It may also be that there is more variation among participants in terms of the common humanity subscale such as due to cultural factors, ethnicity, gender or age. Nevertheless, other subscales and the overall BCQ showed excellent validity. Indeed, that motivated action was not associated with most of the subscales (beyond common humanity) of an existing measure of body compassion (the BCS) but did contribute to the overall score of the measure developed here (the BCQ), suggests that the BCQ includes self-compassionate processes, specifically the second psychology, that are not included in the BCS.

Another issue is with the differences between the subsample included in the test-retest phase. These participants were older and with different BMI averages to that of the larger EFA and CFA samples from which the subsample was retained. There is also an issue with the uptake for the test-retest being quite low, although this was not low enough that the test-retest reliability could not be computed, a higher uptake could improve the generalisability.

Another key issue with studies 2 and 3 is the limited sample sizes. As described in more detail earlier, most associations in studies 2 and 3 had sufficient or moderate power, particularly for body kindness and the overall BCQ score. However, results involving common humanity were generally underpowered. Nevertheless, these studies are indicative of the scale’s validity and add interesting preliminary findings which future research can explore further. Further evaluation of the BCQ is also needed in more specific groups, such as in clinical settings, and with more diverse samples. In addition, while these studies suggested associations with eating disorders and mood, the longitudinal effects of the BCQ might usefully be considered in order to determine its causal association (if any) with various health-related outcomes.

### Implications

Future research can benefit from the addition of the BCQ, a compassion-based rather than MAB-based measure for body compassion that includes components from Gilbert’s (2009, 2010, 2017b), Jazaieri et al.’s (2013) and Neff’s (2003a, 2003b) definitions of compassion and self-compassion. Future research should seek to develop this measure further by testing it in additional groups as detailed above and exploring its relations to health-related behaviours (e.g. physical activity, healthy eating), wellbeing and body image as well as with clinical and non-clinical groups.

The BCQ brings together research from compassion (Gilbert, 2017b), self-compassion (Neff, 2003a) and body-related emotion, distress and feelings. Body compassion has been suggested to explain the relationship between self-compassion and body image threats (Tylka & Wood-Barcalow, 2015) and emerges in interviews when individuals discuss their bodies (Clancy, 2010; Smith, 2013). It is anticipated that the addition of a compassion-informed measure of body compassion might help to facilitate research into relevant domains such as the role of body shame in eating disorders (Troop & Redshaw, 2012), depression (Andrews, 1997) and caloric intake (Troop, 2016) as well as the links between body image in relationship satisfaction (Willis, Palermo, & Burke, 2011), disability (Farhat-ul-Ain & Fatima, 2016), physical activity and well-being (Magnus, Kowalski, & McHugh, 2010).

It will be important to develop models of the role of body compassion in health outcomes. In testing these, the degree to which the body compassion construct is useful over and above general self-compassion is an empirical question but the answer will inform the development of appropriate interventions. Nevertheless, with an increasing range of interventions being developed to increase self-compassion in relation to body image and eating disorders, the BCQ may be an important tool to evaluate outcomes.

## Conclusion

The BCQ has been shown to be a valid and reliable measure of body compassion which taps into aspects of self-compassion in relation to body image that are not included in other similar measures. It is hoped that the development of this measure will encourage additional research into body compassion and facilitate investigations into the relationships between compassion and wellbeing.

## Supplementary Material

Supplemental Material
