# Supplementary material for: Development and validation of the Body Compassion Questionnaire
Source: Health Psychol Behav Med. 2021 Nov 21;9(1):951–88. doi: 10.1080/21642850.2021.1993229 (PMC8635608; doi:10.1080/21642850.2021.1993229)
Supplement: Supplemental Material [file RHPB_A_1993229_SM1954.docx]

Supplementary materials

Table 1 The Body Compassion Questionnaire items

|  |  | To the left of each item, indicate how often you behave or feel in the stated manner, using the following scale: | Almost Never | |  | Almost Always | | |
| --- | --- | --- | --- | --- | --- | --- | --- | --- |
|  |  |  | 1 | 2 | 3 | | 4 | 5 |
| 1 | *BK* | I like my body in spite of small inadequacies |  |  |  | |  |  |
| 2 | *CH* | Body image is something that most people have issues with |  |  |  | |  |  |
| 3 | *MA* | I am trying to become more accepting of my body |  |  |  | |  |  |
| 4 | *CH* | Everyone has mixed feeling about their body |  |  |  | |  |  |
| 5 | *BK* | I do not really think a lot about my body, I accept this is me |  |  |  | |  |  |
| 6 | *CH* | Everyone has something they do not like about their body |  |  |  | |  |  |
| 7 | *BK* | I accept the flaws in my body, even if I don’t like them |  |  |  | |  |  |
| 8 | *MA* | I try my best to accept my body |  |  |  | |  |  |
| 9 | *BK* | I have stopped worrying about weight and body shape |  |  |  | |  |  |
| 10 | *CH* | I am sure everyone has insecurities about their bodies |  |  |  | |  |  |
| 11 | *MA* | I try to be kind to myself about my body |  |  |  | |  |  |
| 12 | *CH* | There are people who have the same or even worse thoughts about their body image than I do |  |  |  | |  |  |
| 13 | *BK* | I am really grateful for the way my body is |  |  |  | |  |  |
| 14 | *MA* | I am working on making myself feel better about the way I look |  |  |  | |  |  |
| 15 | *CH* | I do not think anyone is completely satisfied with their body |  |  |  | |  |  |
| 16 | *BK* | I am thankful for the way I look |  |  |  | |  |  |
| 17 | *CH* | Nearly everyone has some negative feelings about their bodies |  |  |  | |  |  |
| 18 | *MA* | I need to be more accepting of my body |  |  |  | |  |  |
| 19 | *BK* | I feel ok with my body the way it is |  |  |  | |  |  |
| 20 | *MA* | I try to empathise with myself and say that I am ok and that I am happy with my body |  |  |  | |  |  |
| 21 | *BK* | I accept my body the way it is and am comfortable in my own skin |  |  |  | |  |  |
| 22 | *CH* | The way I feel about my body is probably a normal thing for everyone |  |  |  | |  |  |
| 23 | *BK* | I am quite comfortable in my body |  |  |  | |  |  |

Columns in italics are not shown to participants. To calculate overall body compassion, a global mean score calculated. To compute subscales scores, all items are summed and then divided by the number of items in each subscale:

BK = body kindness, expressing kindness and understanding towards one’s body, without criticism or judgement. Includes elements of gratitude, acceptance and comfort in one’s own skin. 1, 5, 7, 9, 13, 16, 19, 21, 23 (9 items)

CH = common humanity, realisation/understanding that one’s feelings are not just experienced by you, but by all humans, are common among peers, family and strangers. 2, 4, 6, 10, 12, 15, 17, 22 (8 items)

MA = motivated action, motivation and actual attempts to change one’s feelings and accept the positive as well as the negative. 3, 8, 11, 14, 18, 20 (6 items)
